# Supplementary material for: Interferon Is Superior to Direct Acting Antiviral Therapy in Tertiary Prevention of Early Recurrence of Hepatocellular Carcinoma
Source: Cancers (Basel). 2019 Dec 19;12(1):23. doi: 10.3390/cancers12010023 (PMC7016942; doi:10.3390/cancers12010023)
Supplement: Supplementary file 1 [file cancers-12-00023-s001.pdf]

cancers

MDPI

Article

Interferon Is Superior to Direct Acting Antiviral Therapy in Tertiary Prevention of Early Recurrence of Hepatocellular Carcinoma

Wei Teng [1,2,3], Wen-Juei Jeng [1,2,3,4,*], Hwai-I Yang [2,5], Wei-Ting Chen [1,3], Yi-Chung Hsieh [1,3], Chien-Hao Huang [1,3], Chen-Chun Lin [1,3], Chun-Yen Lin [1,3,*], Shi-Ming Lin [1,3,4] and I-Shyan Sheen [1,3]

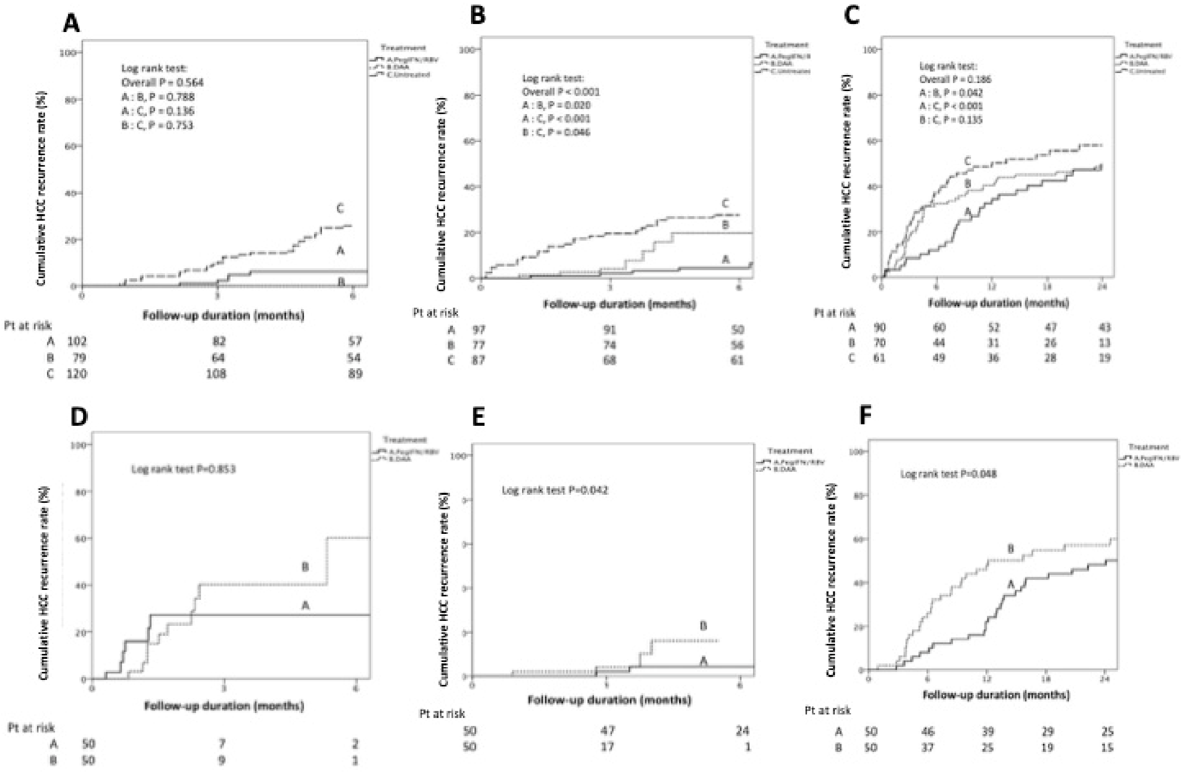

Supplementary Figure S1. Kaplan-Meier curves showing cumulative recurrence rate at different time frames. (**A**): among untreated, DAA and Peg-IFN/RBV arm in Frame I before PSM; (**B**): among untreated, DAA and Peg-IFN/RBV arm in Frame II before PSM; (**C**): among untreated, DAA and Peg-IFN/RBV arm in Frame III before PSM; (**D**): between DAA and Peg-IFN/RBV arm in Frame I after PSM; (**E**): between DAA and Peg-IFN/RBV arm in Frame II after PSM; (**F**): between DAA and Peg-IFN/RBV arm in Frame III after PSM. Supplementary.

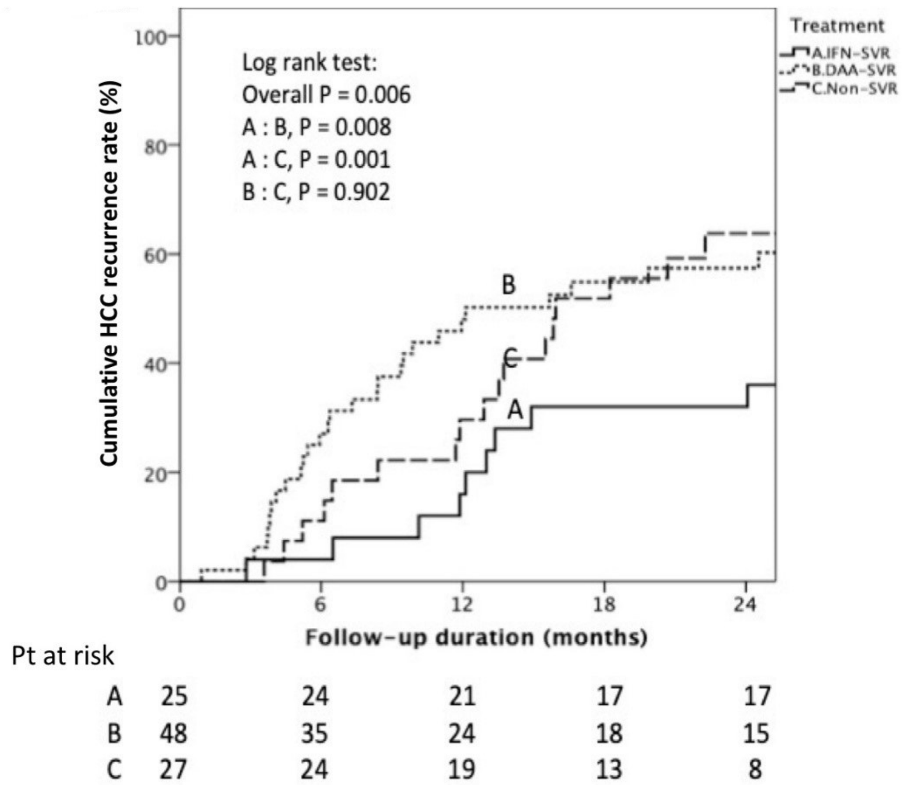

Supplementary Figure S2. Kaplan-Meier curves showing cumulative recurrence rate (**A**): among SVR, non-SVR and untreated patients from the start of antiviral therapy before PSM; (**B**): among DAA- SVR, IFN-SVR, non-SVR and untreated patients from the start of antiviral therapy before PSM; (**C**): among DAA- SVR, IFN-SVR and non-SVR from the start of antiviral therapy after PSM.

Supplementary Table S1. Characteristics comparison among untreated, Peg-IFN/RBV and DAA arms before PSM.

Variables	Overall (*N* = 301)	PegIFN/RBV (*N* = 102)	IFN-free DAAs (*N* = 79)	Untreated (*N* = 120)	*p* value
**Time of hepatoma treatment**					
Age (years) *	65.4 ± 10.2	59.3 ± 9.0	67.1 ± 8.5	69.5 ± 9.8	<0.0001
Gender (male, %)	159 (52.8)	56 (54.9)	38 (48.1)	65 (54.2)	0.6247
TNM stage I/II/III, *n* (%)	191/87/23 (63.5/28.9/7.6)	72/24/6 (70.6/23.5/5.9)	54/22/3 (68.4/27.9/3.7)	65/41/14 (54.2/34.2/11.6)	0.0577
HCC treatment, *n* (%) Resection/RFA/Others	59/135/107(19.6/44.9/35.5)	46/36/20(45.1/35.3/19.6)	13/45/21 (16.5/57.0/26.5)	0/54/66(0/45.0/55.0)	<0.0001
Total bilirubin (mg/dL)	0.7 (0.2–2.9)	0.7 (0.3–2.8)	0.7 (0.2–2.9)	0.8 (0.3–2.5)	0.8251
ALT (U/L)	64 (8–372)	80 (15–372)	74 (19–202)	46 (8–251)	<0.0001
Albumin (g/dL) *	3.74 ± 0.49	3.88 ± 0.55	3.76 ± 0.41	3.61 ± 0.46	<0.0001
AFP (ng/mL)	21 (2-33163)	16 (3–32539)	22 (2–6260)	25 (2–33163)	0.3651
Platelet (10^3/μL)	109 (15–436)	126 (17–393)	84 (33–217)	104 (15–436)	<0.0001
CTP class A/B, *n* (%)	273/28 (90.7/9.3)	94/8 (92.2/7.8)	76/3 (96.2/3.8)	103/17 (85.8/14.2)	0.0414
ALBI grade I/II+III, *n* (%)	124/177 (41.2/58.8)	50/52 (49.0/51.0)	28/51 (35.4/64.6)	46/74 (38.3/61.7)	0.0060
FIB-4	5.56 (0.70–40.9)	3.99 (0.70–19.5)	6.65 (2.16–22.3)	5.92 (0.96–40.9)	<0.0001
APRI	1.92 (0.21–21.7)	1.76 (0.27–11.7)	2.09 (0.52–10.6)	1.71 (0.21–21.7)	0.0398
Tumor numbers, *n* (%)	1 (1–5)	1 (1–5)	1 (1–5)	1 (1–3)	0.5889
Target lesion size (cm)	2.4 (0.7–10.2)	2.5 (0.8–10.0)	2.0 (0.7–5.2)	2.8 (0.8–10.2)	<0.0001
F/u since HCC CR (months)	53.6 (3.4–224.8)	91.3 (3.4–224.8)	53.4 (4.9-190.2)	35.9 (3.8–100.8)	<0.0001
**Time of HCV treatment**	**Overall (*N* = 174)**	**PegIFN/RBV (*N* = 97)**	**IFN-free DAAs (N=77)**		
Age (years) *	64.6 ± 9.9	60.9 ± 9.3	69.3 ± 8.5		<0.0001
Total bilirubin (mg/dL)	0.9 (0.2–5.7)	0.8 (0.3–5.7)	1.0 (0.2–4.2)		0.0418
ALT (U/L)	92 (15–349)	111 (16–349)	75 (15–239)		0.0005
Albumin (g/dL) *	3.93 ± 0.50	3.93 ± 0.51	3.94 ± 0.49		0.8581
AFP (ng/mL)	14 (2–754)	14 (2–754)	14 (2–405)		0.6375
Platelet (10^3/μL)	111 (23–393)	126 (57–393)	93 (23–233)		0.0003
ALBI grade I/II+III, *n* (%)	97/77 (55.8/44.2)	55/42 (56.7/43.3)	42/35 (54.6/45.4)		0.7262
FIB-4	5.72 (0.70–32.8)	4.48 (0.70–18.2)	7.99 (1.39–32.8)		<0.0001
APRI	2.43 (0.29–16.0)	2.28 (0.29–16.0)	2.73 (0.45–12.4)		0.1096
Genotype I, *n* (%)	130 (74.7)	63 (65.0)	67 (87.0)		0.0009
Time to HCV treatment (months)	6.3 (0.1–141.8)	6.1 (0.1–110.6)	8.2 (0.4–141.8)		0.5216
SVR, *n* (%)	130 (74.7)	56 (57.7)	74 (96.1)		<0.0001
F/u since HCV treatment (months)	39.7 (4.0–193.2)	72.5 (9.7–193.2)	29.3 (4.0–53.6)		<0.0001

* Demonstrated as mean ± standard deviation. Abbreviations: AFP, alpha-fetoprotein; ALBI, albumin-bilirubin; ALT, alanine aminotransferase; APRI, AST to platelet ratio index; CR, complete response; CTP, Child-Turcotte-Pugh; DAA, direct acting antiviral agents; FIB-4, fibrosis-4; F/u, follow-up; HCC, hepatocellular carcinoma; HCV, hepatitis C virus; PegIFN/RBV, pegylated interferon plus ribavirin; RFA, radiofrequency ablation; SVR, sustained virologic response.

Supplementary Table S2. HCC recurrence pattern comparison between Peg-IFN/RBV and DAA arms before PSM.

Recurrence before initiation of HCV Tx	Overall (*N* = 39)	PegIFN/RBV (*N* = 17)	IFN-free DAAs (*N* = 22)	*p* value
Type of recurrence, *n* (%)				0.100
Local recurrence	11 (28.2)	6 (35.3)	5 (22.7)	
Intrahepatic metastasis	28 (71.8)	11 (64.7)	17 (77.3)	
Tumor numbers, *n* (%)				
Less than three	35 (89.7)	14 (82.4)	21 (95.5)	0.300
Maximum tumor size	1.7 (0.7-4.0)	1.2 (0.7-4.0)	1.9 (1.1-3.8)	0.037
Vascular invasion	3 (7.7)	3 (17.6)	0 (0)	0.074
Distant metastases	1 (2.6)	1 (5.9)	0 (0)	0.436
**Recurrence after initiation of HCV Tx to post-EOT 2 years**	**Overall (*N* = 96)**	**PegIFN/RBV (*N* = 50)**	**IFN-free DAAs (*N* = 46)**	***p* value**
Type of recurrence, *n* (%)				0.112
Local recurrence	40 (41.7)	17 (34.0)	23 (50.0)	
Intrahepatic metastasis	56 (58.3)	33 (66.0)	23 (50.0)	
Tumor numbers, *n* (%)				
Less than three	70 (72.9)	37 (74.0)	33 (71.7)	0.803
Maximum tumor size	1.8 (0.3–10.3)	1.8 (0.7-4.0)	1.7 (0.3-10.3)	0.880
Vascular invasion	6 (6.3)	3 (6.0)	3 (6.5)	0.916
Distant metastases	6 (6.3)	6 (12.0)	0 (0.0)	0.015

Abbreviations: DAA, direct acting antiviral agents; HCV, hepatitis C virus; PegIFN/RBV, pegylated interferon plus ribavirin; Tx, treatment.

Supplementary Table S3. Predictors for HCC recurrence in Peg-IFN/RBV treated and DAA treated patients before PSM by Cox regression analysis.

Variables		Crude HR	95% CI	*p* value	Adjusted HR	95% CI	*p* value
**IFN-overall (*N* = 97)**							
TNM	I	Referent					
	II/III	1.594	0.887–2.865	0.1192			
Previous recurrence history	No	Referent					
	Yes	2.584	1.274–5.243	0.0094			
**IFN-SVR (*N* = 56)**							
No risk factors							
**IFN-non-SVR (N=41)**							
TNM	I	Referent					
	II	2.282	0.944–5.514	0.0671			
Interval between HCC and HCV	≤8 mo	Referent					
	>8 mo	0.579	0.224–1.495	0.2584			
Previous recurrence history	No	Referent					
	Yes	4.210	1.307–13.56	0.0167			
**DAA-overall (*N* = 77)**							
AFP	≤20	Referent					
	>20	1.452	0.812–2.595	0.2091			
ALBI grade	I	Referent			Referent		
	II/III	2.255	1.248–4.076	0.0072	2.374	1.310–4.301	0.0041
FIB-4	≤6.25	Referent					
	> 6.25	1.636	0.872–3.070	0.1256			
APRI	≤2	Referent					
	>2	1.842	0.969–3.503	0.0630			
Genotype	I	Referent			Referent		
	Non-I	2.590	1.239–5.411	0.0113	2.828	1.352-5.913	0.0064
Interval between HCC and HCV	≤8 mo	Referent					
	>8 mo	0.617	0.343–1.111	0.1082			
**DAA-SVR (*N* = 74)**							
AFP	≤20	Referent					
	>20	1.544	0.859–2.777	0.1471			
ALBI grade	I	Referent			Referent		
	II/III	2.065	1.139–3.745	0.0174	2.187	1.202-3.979	0.0101

FIB-4	≤6.25	Referent					
	>6.25	1.725	0.904–3.293	0.0982			
APRI	≤2	Referent					
	>2	1.956	0.989–3.794	0.0670			
Genotype	I	Referent			Referent		
	Non-I	2.498	1.191–5.237	0.0152	2.731	1.301–5.733	0.0082
Interval between HCC and HCV	≤8 mo	Referent					
	>8 mo	0.585	0.322–1.063	0.0798			

Abbreviations：DAA, direct acting antiviral agents; FIB-4, Fibrosis-4; PegIFN/RBV, pegylated interferon plus ribavirin; SVR, sustained virologic response;.

Supplementary Table S4: Characteristics comparison at time of antiviral therapy between HCC recurrent and non-recurrent patients.

**Variables**	**Overall (*N* = 100)**	**Recurrence (*N* = 53)**	**Non-recurrence (*N* = 47)**	***p*-value**
Age (years) *	65.8 ± 8.1	66.0 ± 8.4	65.6 ± 7.8	0.7651
Gender (male, %)	52 (52.0)	27 (50.9)	25 (53.2)	0.8436
TNM stage I/II/III, *n* (%)	71/26/3 (71.0/26.0/3.0)	35/17/1 (66.0/32.1/1.9)	36/9/2 (76.6/19.2/4.2)	0.3242
HCC treatment, *n* (%) Resection/RFA/Others	24/52/24 (24.0/52.0/24.0)	10/30/13 (18.9/56.6/24.5)	14/22/11 (29.8/46.8/23.4)	0.4343
Total bilirubin (mg/dL)	0.8 (0.4–4.1)	1.1 (0.4–4.1)	0.7 (0.4–2.4)	0.0193
ALT (U/L)	86 (16–300)	83 (16–290)	91 (27–300)	0.5853
Albumin (g/dL) *	3.95 ± 0.51	3.81 ± 0.48	4.10 ± 0.50	0.0045
AFP (ng/mL)	14 (2–317)	21 (3–317)	10 (2–265)	0.0118
Platelet (10^3/μL)	114 (31–251)	94 (31–251)	121 (42–219)	0.0490
ALBI grade I/II+III, *n* (%)	57/43 (57.0/43.0)	22/31 (41.5/58.5)	35/12 (74.5/25.5)	0.0016
FIB-4	5.63 (0.97–26.1)	6.56 (0.97–26.1)	4.64 (1.39–16.4)	0.0203
APRI	2.44 (0.30–16.0)	2.65 (0.30–16.0)	2.11 (0.45–12.3)	0.1076
Antiviral treatment				
PegIFN/RBV, *n* (%)	50 (50.0)	24 (45.3)	26 (55.3)	0.4230
DAA, n (%)	50 (50.0)	29 (54.7)	21 (44.7)	
SVR, *n* (%)	73 (73.0)	36 (67.9)	37 (78.7)	0.2639
Non-SVR, *n* (%)	27 (27.0)	17 (32.1)	10 (21.3)	
Time to HCV treatment (months)	7.2 (0.1–141.8)	5.1 (0.2–110.6)	12.8 (0.1–141.8)	0.0079
Genotype 1, *n* (%)	75 (75.0)	40 (75.5)	35 (75.5)	1.0000
Tumor numbers, *n* (%)	1 (1–5)	1 (1–5)	1 (1–5)	0.2687
Target lesion size (cm)	2.0 (0.8–10.0)	2.0 (0.8–10.0)	2.0 (0.8–5.2)	0.9862
F/u since HCV treatment (months)	40.1 (11.7–184.1)	36.5 (13.3–119.9)	50.6 (11.7–184.1)	0.1403

* Demonstrated as mean ± standard deviation. Abbreviations: AFP, alpha-fetoprotein; ALBI, albumin-bilirubin; ALT, alanine aminotransferase; APRI, AST to platelet ratio index; CR, complete response; DAA, direct acting antiviral agents; FIB-4, fibrosis-4; F/u, follow-up; HCC, hepatocellular carcinoma; HCV, hepatitis C virus; PegIFN/RBV, pegylated interferon plus ribavirin; RFA, radiofrequency ablation; SVR, sustained virologic response.
